# Nutritional, Medicinal and Toxicological Attributes of Star-Fruits (Averrhoa carambola L.): A Review

**DOI:** 10.6026/97320630012420

**Published:** 2016-12-22

**Authors:** Narmataa Muthu, Su Yin Lee, Kia Kien Phua, Subhash Janardhan Bhore

**Affiliations:** 1 Department of Biotechnology, Faculty of Applied Sciences, AIMST University, Bedong-Semeling Road, Semeling 08100, Kedah,Malaysia; 2 Institute for Research in Molecular Medicine (INFORMM), Universiti Sains Malaysia, 11800, Penang, Malaysia;

**Keywords:** Averrhoa carambola L., medicinal plants, nutrition, oxalic acid, star-fruits, toxicity

## Abstract

Plants are very complex organisms that produce medicinally important natural products. The Star-fruit producing plant (Averrhoa carambola L.) is a species of woody plant in the family Oxalidaceae native to the Philippines, Indonesia, Malaysia, Vietnam, India, Bangladesh and Sri Lanka; but, cultivated in many parts of the world. Star-fruits are popular tropical fruits and used commonly in Ayurvedic and Traditional Chinese Medicines (TCM) in India, China, and Brazil to relieve ailments such as chronic headache, fever, cough, gastro-enteritis, diarrhoea, ringworm infections, and skin inflammations. However, this fruit contains high amount of oxalate, which is hazardous for uremic patients, and caramboxin (CBX), which is neurotoxic. The aim of this review is to highlight the nutritional, medicinal and toxicological traits of the star-fruits.

## Background

The Star-fruit plant (family: Oxalidaceae; species: Averrhoa carambola L.) is widely distributed around the world, especially in tropical countries such as India, Malaysia, Indonesia, and Philippines. This Star-fruit plant belongs to the genus, Averrhoa, which contains 5 species, namely A. bilimbi, A. dolichocarpa, A. leucopetala, A. microphylla and A. carambola. However, A. carambola is widely cultivated on a commercial scale [1]. Averrhoa carambola is considered the most important species and cultivated extensively in South-east Asia and Malaysia [2,3]. In addition, it is a popular fruit in the United States, Australia and South Pacific Islands market [4] . Star-fruits are fleshly, crunchy, juicy and slightly tart, acidic and sweet in the taste. This fruit is known to have high antioxidant property that efficiently scavenge free radicals as well as helps in hypoglycemic and hypocholesterolemia treatments [5, 6,7]. Star-fruits are also commonly used in preparation of juice, pickles and salads. However, it can be eaten raw and used for cleaning utensils; because, it helps in removing the rust caused by iron oxidation. Star-fruits are well known for the oxalic acid content in it which gives an adverse effect when consumed by uremic patients [8, 9,10]. The aim of this review article is to highlight the nutritional, medicinal and toxicological attributes of the Star-fruit.

## Methodology

### Botanical description

**Figure 1:**
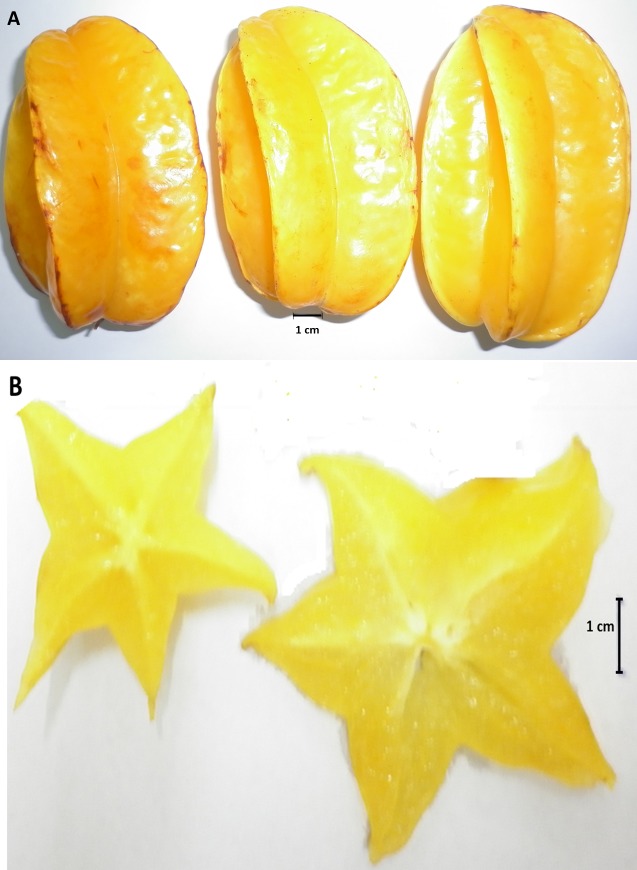
Appearance and colour of the fully ripen Star-fruits (Averrhoa carambola L.). A) Fully ripen Star-fruits showing longitudinal ridges; B) star-shaped slices of fruits when cut horizontally; bar shows the scale of 1 centimetre.

Averrhoa carambola is a slow growing species of woody plants; it is multi-stemmed with short trunk and best grows up to 6 to 9 m in height. It has a bushy appearance with many branches producing a broad, rounded crown and a trunk base which can reach up to 15cm in diameter [3,11]. In addition, the tree has leaflets that fold together at nightfall and shows sensitivity to light and shock, such as abrupt movements of the leaves. Star-fruits plant produces small clusters of red, lilac or purple flowers containing five petals. Fruits are usually small and dark green in colour when unripe; but, fruits turn yellow in colour when they are fully ripe. Usually, star-fruits are fleshy with 5 longitudinal ridges or angles (Figure 1A), and are crunchy crisp in texture. Furthermore, the fruits are star-shaped when cut horizontally (Figure 1B); hence, the fruit is called as Star-fruit [12,13]. The firmness and colour of the star-fruits changes with its development as shown in Table 1. The taxonomical classification of Star-fruit plant and common names of star-fruits are given in Table 2and 3, respectively.

### Star-fruit plant varieties

In Malaysia, A. carambola is a commercial cultivar, and its fruits are widely marketed in all the states and exported mainly to the Europe. In Malaysia, four states namely, Selangor, Negeri Sembilan, Pahang, and Johor are cultivating Star-fruits [14]. Nineteen (19) varieties of star-fruit are registered under the Department of Agriculture, Malaysia. However, out of these 19 varieties, only two varieties are popular as the best commercial clones, namely ‘Belimbing Besi’ (B10) and ‘Belimbing Madu’ (B17) [15,16]. Besides Malaysia, the United States (USA) also cultivates Star-fruit plants for its fruits [3]. Taiwan has its own collection of Star-fruit plant accessions, such as ‘Mih Tao’, ‘Dah Pon’, ‘Tean Ma’ and in Thailand, ‘Fwang Tung’[3] . Registered and widely accepted superior clones (varieties) of the Star-fruit plants are depicted in the Table 4.

### Nutritional attributes of star-fruits

The star-fruit is a good source of various minerals and vitamins (Table 5). Star-fruits are also a rich source of natural antioxidants such as L-ascorbic acid (Vitamin C) and Gallic acid, which aid in scavenging reactive oxidative species (ROS) [17]. The literature shows that Star-fruits are a good source of magnesium, potassium, phosphorous, as well as β-carotene, and vitamin C, which are common antioxidants [18]. The presence of antioxidants like iron, zinc and manganese in the fruits aid in strengthening the immune system [19]. In addition, the presence of high amounts of fibres in fruits aids in absorbing glucose and retarding the glucose diffusion into the blood stream; as a result, it helps in controlling blood glucose concentration [20]. The Starfruit intake also exhibits hypo-cholesterolemic and hypolipidaemic effect as it enhances the removal of cholesterol, lipid, and bile acid through the excrement [21].

### Medicinal properties

Nowadays, herbal medications are becoming popular worldwide as an alternative therapy to drug medication. In addition to food source, Star-fruits are also considered as herbs in many parts of Brazil, China, India, and Malaysia as well as in Taiwan, and widely used in Ayurvedic and traditional Chinese Medicine [22, 23] preparations as remedy for fever, sore throat, cough, asthma, chronic headache, and skin inflammations. The phytochemical and pharmacological studies suggest that the extracts of Star-fruit plant leaves, fruits and roots contains saponins, flavonoids, alkaloids and tannins [24, 25] which are known to confer antioxidant and specific healing properties [26]. The major vitamins and acids found in star-fruits are highlighted in Table 6.

### Antioxidant property

Studies reported that Star-fruits contain proanthocyanins which serves as an antioxidant besides Vitamin C and Gallic acid [7] . The main purpose of antioxidants is to scavenge ROS such as peroxides. Usually, fatty acids are susceptible to oxidative damage by peroxides and hyperperoxides [27]. Consumption of Star-fruits is helpful in removing toxins from the body and aids the immune system in guarding against cancer, ROS damage and lipoperoxidation [28].

### A source of water insoluble fibres

Usually, when consuming Star-fruit juice, often the fibre’s residual parts of fruits are excluded from the beverage. In spite of this, Star-fruit contains approximately 60% of cellulose, 27% of hemicelluloses and 13% of pectin [29]. It indicates that star-fruit is indeed rich in insoluble fibres fractions (IFF). The insoluble fibres have the ability to retain water more than cellulose; thus called as ‘water insoluble fibre fractions’ or WIFF. WIFFs actually aids in smooth movement of the bowels and has the capability of lowering blood glucose by slowing down the absorption of carbohydrate in our body [29,30]. In addition, the fibres also facilitate in lowering the total cholesterol level in the body by promoting hypoglycaemic effect. Consuming the fruit-juice together with the fibres (called as smoothie) does help in removing lipids through the excrement, and thus lowering the risks of cardiovascular diseases. It has also been reported that Star-fruit extracts do have selective anti-brain-tumour activity [31,32].

### Anti–inflammatory and anti–microbial property

Research findings of Cabrini et al [17, 33] indicate that antiinflammatory activity of Star-fruit extracts help in lowering the skin inflammatory condition. Researchers induced a skin inflammatory condition akin to eczema using croton-oil on a mice model. When ethanolic extracts of Star-fruit plant leaves were applied on the skin, it resulted in reduced inflammation and gradually reduced eczema in the mice [8]. In addition to this, the extracts in various concentrations were found to inhibit the growth of Staphylococcus aureus (MBC of 15.62mg/ml) and Klebsiella spp. (MBC of 125mg/ml) [9]. Extracts were also effective against Escherichia coli, Pseudomonas aeruginosa and Bacillus cereus [8,10,34].

### Anti-ulcer property

Traditionally, star-fruits are used to relieve stomach discomfort or any ulcer-like disorders. The research findings of Cabrini et al [17, 33] demonstrated that extracts of Star-fruit plant leaves have anti-ulcerogenic properties. The extracts contain terpenoids (diterpenes and triterpenes), flavonoids and mucilage, which are known to have the anti-ulcer activity. The mucilage provides a lining to the gastro-intestinal mucosa, thus helping to avoid damages due to gastritis [35].

### Toxicological effects

Star-fruits do possess many magnificent properties. However, this fruit also poses threat to health as it exudes toxic effects in high uremic patients or patients with chronic renal disease due to its high oxalate content [36,37]. Patients with renal disease are unable to secrete toxic substances out of their body efficiently; as a result of it, they are affected adversely by the oxalates [38]. The first toxicological effect was demonstrated on mice model by Muir and Lam [39]. Variable dosages of the fruit extracts were prepared and injected into the mice through intra-peritoneal injection, and fruit extracts exceeding 8g/kg provoked convulsions and death in the mice [35]. Further analysis of the test reports showed that Star-fruit juice with oxalate content was responsible for the death of rats. Chronic renal failure patients had high mortality rate after consuming the Star-fruits [40,41]. It was noted that these patients had symptoms of hiccups, mental confusions or disturbance in consciousness and vomiting before succumbing to death. Reports also suggest that uraemic patients experienced nephrotoxic and neurotoxic effects when they consumed Star-fruit [42]. Most of the patients were able to recover after immediate hemodialysis, which spanned for weeks but some experienced total renal failure, causing death. Even though star-fruits have many documented nutritional and medicinal benefits; but, due to the oxalate and caramboxin content in the fruits, it is considered toxic to patients experiencing renal problems.

### Perspective

Indisputably, fruits are very important in our daily diet for various health benefits. However, some fruits may contain high amounts of unique secondary metabolites, which are hazardous to our health. Star-fruit plants are cultivated commercially in tropical countries for their fruits. This fruit have several medicinal properties; hence, it is used medicinally for many years in Ayurvedic treatments. Star-fruits contain various antioxidants which are considered medicinally important and beneficial for the health. However, the negative part of this fruit is that it produces oxalic acid and caramboxin, which are toxic to uremic patients. It can cause death if consumed in sufficient quantities by those experiencing renal failure. Thus, more public awareness about oxalate poisoning on uremic patients should be promoted. It will help to avoid adverse side effect of star-fruits in high uremic patients. It is very important that the public is well educated on the benefits as well as the hazardous effects of the star-fruits.

Modern techniques and molecular biology knowledge should be utilized to understand gene expression in star-fruits. A systematic ‘transcriptomics’ study of the star-fruit will help us to elucidate the genes expressed in it, and the genes involved in biosynthesis of oxalic acid. Furthermore, transcriptomics study will be helpful to discover the novel genes expressed in the star-fruits. If we identify and characterize the key genes involved in oxalic acid and CBX biosynthesis, then the genetic alteration of the Star-fruit genome can be considered as one of the strategies to knock-out the oxalic acid and CBX synthesis in fruit specific manner. Various post-transcriptional gene silencing (PTGS) techniques such as antisense, inverted-repeats and or intron-spliced inverted repeats [43] can be utilized in genetic engineering of the Star-fruit plant to knock-out the biosynthesis of oxalic acid and CBX. If we develop genetically engineered Star-fruit plants which will produce fruits devoid of oxalic acid and CBX then it will certainly help to increase not only its economic value but nutritional value also.

## Conclusion

Star-fruit is a good source of nutritionally and medicinally important natural products beneficial for human health. However, due to the oxalate and caramboxin content in the fruits, it is toxic to patients with renal problems. Transcriptomics study of the Star-fruit will help us to understand genes expressed in it and the discovery of novel genes could help in designing a novel strategy for genetic engineering of this plant to knock-out the oxalate and caramboxin biosynthesis in fruit-specific manner to enhance its nutritional quality.

## Disclosure

Authors attest that there are no conflicts of interest to declare.

## Figures and Tables

**Table 1:** The developmental stages, firmness and colour of Averrhoa carambola L. fruits

Stage	Firmness	Colour
Young	Firm Texture	100% green colour
Half-Ripe	Firm Texture	Yellowish green
Ripe	Soft Texture	100% Yellow colour

**Table 2:** Taxonomical classification of Star-fruit plant

Taxonomy	Classification
Division:	Spermatophyta
Sub-Division:	Angiospermae
Class:	Dicotyledonae
Sub-Class:	Polypetalae
Series:	Disciflorae
Order:	Geraniales
Family:	Oxalidaceae
Genus:	Averrhoa
Species:	carambola

**Table 3:** Common names of Star-fruit (Averrhoa carambola L.) in different dialect

Language	Name
Bengali	Kamranga
English	Star-fruit, Chinese Gooseberry
Haiti	Zibline
Hindi	Kamrakh
Indonesian	Blimbing
Malay	Belimbing
Tamil	Thambaratham
Sanskrit	Kamaranga
Venezuela	Tamarindo

**Table 4:** Registered varieties of Star-fruits plant, Averrhoa carambola L.

No	Variety Registry No	Variety name	Year Registered	Country
1	B1	Yong Toh Yin	1937	Malaysia
2	B2	MAHA 66	1966	Malaysia
3	B3	Foo Red	1966	Malaysia
4	B4	Sg.Besi 1	1966	Malaysia
5	B5	Sg. Besi 2	1966	Malaysia
6	B6	Sg. Besi 3	1966	Malaysia
7	B7	Sg. Besi 4	1966	Malaysia
8	B8	Sg. Besi 5	1968	Malaysia
9	B9	NA	1968	Malaysia
10	B10	Belimbing Besi *	1968	Malaysia
11	B11	Chan Yong 1	1968	Malaysia
12	B12	Belimbing Madu Chan Yong 2*	1968	Malaysia
13	B13	Istana Perak 1	1971	Malaysia
14	B14	Istana Perak 2	1971	Malaysia
15	B15	Istana Perak 3	1971	Malaysia
16	B16	NA	NA	Malaysia
17	B17	Belimbing Madu*	1988	Malaysia
18	B18	NA	1989	Malaysia
19	B19	NA	1989	Malaysia
20	NA	Golden Star*	1965	USA
21	NA	Newcomb	1965	USA
22	NA	Thayer	1965	USA
23	NA	Arkin	1965	USA
24	272065	Mih Tao*	1963	Taiwan
25	NA	Dah Pon	1963	Taiwan
26	NA	Tean Ma	1963	Taiwan
27	NA	Fwang Tung*	1973	Thailand

*indicates the commercially cultivated superior clones of Star-fruit plant; NA, not available; USA, United States.				

**Table 5:** Minerals found in the star-fruits (Averrhoa carambola L.) [12]

Mineral	Amount [mg/100g fruit]*
Sodium (Na)	3.8 - 3.85
Potassium (K)	167.13 - 168.0
Calcium (Ca)	6.37 - 6.40
Phosphorous (P)	17.87 - 17.88
Magnesium (Mg)	11.85 - 12.05
Iron (Fe)	0.34 - 0.45
Copper (Cu)	0.19 - 0.45
Zinc (Zn)	0.29 - 0.51
Manganese (Mn)	0.04 - 0.52
Selenium (Se)	Not Detectable

*on a dry weight basis	

**Table 6:** Carotene, vitamins and acids found in mature star-fruits (Averrhoa carambola L.)

Name	Amount [mg/100g Star-fruit weight]*
Carotene	0.003 - 0.55
Tartaric acid	4.37
Oxalic acid	9.6
Ketoglutaric acid	2.2
Citric acid	1.32
Vitamin B1 & B2	0.12
Vitamin C	25.8

*on a dry weight basis	

## Abbreviations

CBX: caramboxin
MBC: minimum bactericidal concentration
PTGS: Post-Transcriptional gene silencing
TCM: Traditional Chinese Medicines
